# Relationship between socioeconomic status and cognitive ability among Chinese older adults: the moderating role of social support

**DOI:** 10.1186/s12939-023-01887-6

**Published:** 2023-04-24

**Authors:** Leiyu Shi, Lei Tao, Nanqian Chen, Hailun Liang

**Affiliations:** 1 Johns Hopkins Primary Care Policy Center, 624 N Broadway, Baltimore, USA; 2 School of Public Administration and Policy, Renmin University of China, 59 Zhongguancun, Beijing, China; 3 Department of Public and International Affairs, City University of Hong Kong, Hong Kong SAR, China; 4 Department of Sociology, The Chinese University of Hong Kong, Hong Kong SAR, China

**Keywords:** Cognitive ability, Socioeconomic status disparities, Social Support

## Abstract

**Background:**

Understanding the causes and pathways of cognitive decline among older populations is of great importance in China. This study aims to examine whether the discrepancy in socioeconomic status (SES) makes a difference to the cognitive ability among Chinese older adults, and to disentangle the moderating role of different types of social support in the process in which SES influences cognition.

**Methods:**

We utilized a nationally representative sample from the 2018 Chinese Longitudinal Healthy Longevity Survey. A cumulative SES score was constructed to measure the combined effect of different socioeconomic statuses on the cognitive ability of the elderly. We further examined the moderating role of two types of social support, including emotional support, and financial support. Hierarchical regression analysis was applied to test the direct effect of SES on cognitive ability, and to investigate the moderating role of social support on the association of the SES with the dependent variables.

**Results:**

The results showed that the higher SES of older adults was significantly associated with better cognitive ability (*β* = 0.52, *p* < 0.001) after controlling for age, sex, marital status, living region, Hukou, health insurance, lifestyle factors, and physical health status. Emotional support and financial support were moderated the relationship between SES score and cognitive ability.

**Conclusion:**

Our results reveal the importance of considering social support in buffering the effects of SES and the associated cognitive ability for aging populations. It highlights the importance of narrowing the socioeconomic gap among the elderly. Policymakers should consider promoting social support to improve the cognitive ability among older adults.

## Background

It is widely acknowledged that the initial cognitive function rapidly declines with advancing age [3]. Consequently, the deterioration in cognition among older adults may significantly lower the quality of life and cause many mental health-related illnesses, including depression, delirium, and Alzheimer’s disease [5, 9]. According to recent statistics from World Health Organization [24], dementia is one of the major leading causes of death and disability among older adults worldwide. China is not exceptional due to its mounting proportion of elderly individuals. In 2015, the prevalence of dementia in China was around 6% among older adults, with an estimated 9.5 million patients [26]. Furthermore, it is projected that the number of people with dementia will increase to more than 35 million by 2050 [23]. Thus, understanding the causes and pathways of cognitive decline among older populations is of great importance in China.

While age is the crucial factor for determining cognitive function, a large number of studies suggested that older adults’ cognitive ability also varied by their socioeconomic status (SES), indicating the existence of possible SES inequalities in cognition. For example, Park found that being female and nonwhite ethnicity were significantly associated with lower cognitive function [19]. In addition, compared with the high SES older adults, older people with the poor economic condition and low educational attainment were more likely to experience cognitive impairment [17, 29]. Previous studies also noticed that older individuals who lived in inadequate housing conditions had worse cognitive ability than those in affluent communities [12, 13]. However, most existing studies on the link between SES and cognitive ability were conducted in the high-income country; very few have focused on the context of China by using a nationally representative older sample. With the rapid economic and societal change, considerable socioeconomic and health disparities across China have been reported [11, 15]. It is thus essential to examine whether the discrepancy in SES makes a difference in the cognitive ability among Chinese older adults.

In addition to investigating the direct link between SES and cognitive ability, understanding the pathway underlying such a relationship would help to elucidate the developmental process of cognition [1, 14]. One possible moderator is social support. Social support has been widely demonstrated as a good factor that moderates the relationship between SES and mental disorders such as depression [8, 16]. However, limited research has examined the process in which SES impacts cognitive ability through social support. Indeed, social support is distributed unequally across different social groups [2, 6]. Individuals from low SES typically have limited social access and receive less social support compared to those in high socioeconomic status [20]. As a result, the lower level of social support in the disadvantaged older adults decreases their cognitive function as they lack the access to engage in social communication and participate in social activity [7, 27]. In other words, the absence of social interaction makes the older population more vulnerable to cognitive problems. Thus, social support may serve as an essential moderator in the relationship between SES and cognitive ability.

From a theoretical perspective, cognitive reserve theory and the stress-buffering hypothesis provided clear reason to expect the roles of sources of social support on cognitive outcomes among the elderly [32, 33]. Receiving social support may stimulate social environment in the development of resistance against cognitive aging, according to the cognitive reserve theory [32]. A stimulating social environment can have favorable effects on brain architecture, which may reduce the frequency of memory complaints. In terms of the stress-buffering hypothesis, it states that more social support may reduce negative responses to stressful experiences and negative subjective self-appraisals of cognition [32, 34]. As increased levels of stress were shown strongly associated with the incidence of dementia [35], the perceived availability of social support may serve as a source of regular brain stimulation [33], enjoyment and relaxation, and contribute to healthy behavior, which may weaken or eliminate the negative perceived stress [34].

Additionally, existing studies primarily understood social support as a holistic concept but failed to distinguish the different sources of social support. Social support is a comprehensive term that includes support accessible to an individual through social ties to other individuals, groups, and the larger community, etc. [28, 37]. Previous research suggested that the association between social support and cognitive ability differed by its sources. For example, Ellwardt et al. [4] found that the protective effect of emotional support was more significant than the instrumental support for older adults. It is thus reasonably expected that the moderating role of social support may depend on its source. Given that older adult may receive social support from various sources, it is meaningful to disentangle the role of different types of social support in the process in which SES influences cognition.

This study aims to examine whether SES disparity is related to cognitive ability among Chinese older adults and to test the moderating role of two types of social support, including financial support and emotional support. This study contributes to the understanding of the SES inequalities and the moderating role of social support on the cognition decline among older populations in China. It also helps to shed light on the implications of pathways to improve older adults’ mental health and narrow the socioeconomic status gap in health inequalities.

## Method

### Data source

This study employed the data from the Chinese Longitudinal Healthy Longevity Survey (CLHLS) 2018, a nationally representative population-based survey of older adults in China. The CLHLS aims to understand the impacts of demographic, family, socioeconomic and environmental risk factors that influence the healthy longevity of the elderly Chinese. It is a publicly available dataset (more detailed information can be found at https://sites.duke.edu/centerforaging/programs/chinese-longitudinal-healthy-longevity-survey-clhls/). The CLHLS 2018 conducted face-to-face interviews with 15,874 individuals with their family members using internationally compatible questionnaires. We included participants aged 60 years or older with complete information related to socioeconomic status, cognitive ability test, social support, and covariates, including age, sex, marital status, living region, Hukou, health insurance, lifestyle factors, and physical health status. All participants with one or more missing values in key variables were excluded. We performed multiple imputation by chained equations (MICE) and repeated the main analyses. We assumed data was missed at random, and results before and after multiple imputation were compared and shown no statistically different. The final sample size of the study was 5,295.

## Measures

### Cognitive ability

The dependent variable in this study is cognitive ability. CLHLS utilized the Chinese version of the Mini Mental State Examination (MMSE) to measure cognitive ability among older adults. It contains 24 items to measure several dimensions of cognitive ability, including the test of calculation, language, orientation, and recall ability [10]. For each question, the correct answer was coded as 1, while the error response was coded as 0. The aggregated cognitive ability score ranges from 0 to 30, with higher scores indicating better cognitive function. We performed analysis to assessed the reliability of the MMSE scale, and the results of Cronbach’s alpha is 0.861.

### Socioeconomic status

Based on the previous study [25], we used four different dimensions to measure socioeconomic status: (1) Schooling: Literate = 1, Illiterate = 0; (2) Occupation: White collar = 1, Non-white collar = 0; (3) Economic Independence: Having Retirement Earnings = 1, No Income = 0; and (4) Residence: Urban = 1, Rural = 0. Considering the cumulative effect of socioeconomic status on the cognitive ability of the elderly, we followed the previous studies [21–23, 25] to aggregate the above items to get a cumulative SES score. The total SES score ranges from 0 to 4, with higher scores indicating a higher socioeconomic status.

### Social support

Social support plays an important role in peoples’ late life and it has been defined as “perception and actuality that one is cared for, has assistance available from other people, and that one is part of a supportive social network” [36]. The level of social support primarily depends on the people’s perceived availability of support and help from others [36]. Following the previous studies [36], we included two types of social support, including emotional support and financial support.

Three items were used to measure emotional support: (1) Having people to talk; (2) Having people to share thoughts; (3) Having people to ask for help. Each item was coded as 0 = nobody, and 1 = spouse/son/daughter/daughter in law/son in law/grandchildren/other relatives/friends/social workers/housekeeper. The total score of emotional support ranges from 0 to 3, with a higher score indicating higher emotional support.

Two items were used to measure financial support: (1) Financial support from the son (2) Financial support from daughter. Each item was recoded as a continuous variable (unit: RMB), and were added in total. The total score of financial support were logarithmized, with a higher score indicating higher financial support.

### Control variables

The rationale of selection of control variables was based on the conceptual framework of determinants of health and related previous evidence [30, 31]. Three types of control variables were selected, including demographic variables, lifestyle, and physical health factors. The demographic variables including age group (categorical variable: 65–74 = 1; 75–84 = 2; 85–94 = 3; > = 95 = 4), sex (dummy variable: male = 0; female = 1), marital status (dummy variable: widowed/divorced/never = 0 married; married = 1), living region (categorical variable: West = 0; Middle = 1; East = 2), Hukou (urban = 1, rural = 0), and having health insurance (yes = 1, no = 0). The lifestyle factors include diet diversity (Not diverse = 0, Diverse = 1), drinking (Ever = 0, Never = 1), smoking (Ever = 0, Never = 1), BMI (Underweight, overweight or obese = 0, Normal = 1), and exercise (Ever = 0, Never = 1). The high dietary diversity was defined as higher than the medium of a dietary diversity score. The score was defined by the sum of the consumption frequencies of various food groups, including vegetables, fruits, legumes, and their products, nuts, meat, eggs, fish, tea, according to the previous study [10].The physical health factors include ADL (the index ranging from 0 to 6), depression (No = 0, Yes = 1), hypertension (No = 0, Yes = 1), diabetes (No = 0, Yes = 1) and stroke (No = 0, Yes = 1). For constructing the ADL index, respondents answered six questions about whether they need help from others in bathing, dressing, going to the toilet, indoor activities, controlling urine and feces, and eating. Each question was assigned as 1 for completely or partially needing help and 0 for no help.

### Statistical analysis

We first applied the OLS regression to identify the direct association between SES score and cognitive ability. These key measures were applied and the internal consistencies were tested by the previous studies [28, 38–41].Then, the moderator variables (emotional support, and financial support) were added to the models. Hierarchical regression analysis was applied to test the direct effect of SES on cognitive ability, and to investigate the moderating role of social support on the association of the SES with the dependent variables. We compared the statistical significance of the model with and without social support included to determine whether it moderates the relationship between cognitive ability and SES. We created an interaction term between SES and social support and tested its significance to examine the moderator role of social support. We estimated four models to predict cognitive function, respectively. In each set of regression analyses, we included covariates in Step 1, SES scores in Step 2, social support in Step 3, and an interaction term (e.g., emotional support × SES) in Step 4. In each model, potentially confounding factors were controlled, including age, sex, marital status, living region, Hukou, health insurance, lifestyle factors, and physical health status. All the analyses were performed by Stata/SE version 14.0. Two-tailed P values less than 0.05 were considered statistically significant.

## Results

### Characteristics of samples

Table 1 describes the sample characteristics. Overall, the participants had a good cognitive ability with an average score of 26.17. With regrading to social support, they reported the highest level of emotional support with a mean score of 1.58. The logarithmized score for financial support was 5.55. The average total score of SES was 1.5. Specifically, more respondents reported that they were literature (56.45%), Non-white collar occupation (88.12%), not economic independent (73.35%) and lived in the urban area (55.39%). As for demographic variables, participants had an average age of 82.49, were more female (53.88%), and nearly half of respondents lived in the eastern region of China (48.54%). The Hukou status of most participants were rural (73.24%). In addition, more than half of the participants reported that their diet was not diverse (52.20%), the BMI was normal (54.88%), and they had no exercise (64.25%). 83.49% and 83.14% of participants stated that they never drink and smoke. Of the participants, most of them declared that they had no hypertension (56.32%), diabetes (89.78%), and stroke (89.82%).

**Table 1:** A statistical description of respondents’ characteristics (n = 5,295)

Characteristics		N = 5,295
**Outcome**		
Cognitive ability	Mean (SD)	26.17 (5.01)
**Social support**		
Financial support (logarithm)	Mean (SD)	5.55 (3.59)
Emotional support	Mean (SD)	1.58 (1.32)
**Social economic status (SES)**		
Schooling		
	Literate, N (%)	2989 (56.45%)
	Illiterate, N (%)	2306 (43.55%)
Occupation		
	White collar, N (%)	629 (11.88%)
	Non-white collar, N (%)	4666 (88.12%)
Economic independence		
	Yes, N (%)	1411 (26.65%)
	No, N (%)	3884 (73.35%)
Residence		
	Urban, N (%)	2933 (55.39%)
	Rural, N (%)	2362 (44.61%)
SES score, 0–4	Mean (SD)	1.50 (1.19)
**Control variables**		
**Social demographic**		
Age, years		
	Mean age at baseline, mean (SD)	82.49 (10.98)
	65–74, N (%)	1508 (28.48%)
	75–84, N (%)	1605 (30.31%)
	85–94, N (%)	1294 (24.44%)
	>=95, N (%)	888 (16.77%)
Sex		
	Male, N (%)	2442 (46.12%)
	Female, N (%)	2853 (53.88%)
Marital status		
	Others, N (%)	2772 (52.35%)
	Married and with spouse, N (%)	2523 (47.65%)
Living region		
	West, N (%)	1166 (22.02%)
	Middle, N (%)	1559 (29.44%)
	East, N (%)	2570 (48.54%)
Hukou		
	Urban, N (%)	1417 (26.76%)
	Rural, N (%)	3878 (73.24%)
Health insurance		
	Yes, N (%)	4700 (88.76%)
	No, N (%)	595 (11.24%)
**Lifestyle**		
Diet diversity		
	Not diverse, N (%)	2764 (52.20%)
	Diverse, N (%)	2531 (47.80%)
Smoking status		
	Ever, N (%)	893 (16.86%)
	Never, N (%)	4402 (83.14%)
Drinking status		
	Ever, N (%)	874 (16.51%)
	Never, N (%)	4421 (83.49%)
BMI		
	Underweight, overweight or obese, N (%)	2389 (45.12%)
	Normal, N (%)	2906 (54.88%)
Exercise		
	No, N (%)	3402 (64.25%)
	Yes, N (%)	1893 (35.75%)
**Health conditions**		
ADL	Mean (SD)	0.33 (0.99)
Depression	Mean (SD)	9.17 (4.13)
Hypertension		
	No, N (%)	2982 (56.32%)
	Yes, N (%)	2313 (43.68%)
Diabetes		
	No, N (%)	4754 (89.78%)
	Yes, N (%)	541 (10.22%)
Stroke		
	No, N (%)	4756 (89.82%)
	Yes, N (%)	539 (10.18%)

### Direct effects of SES on cognitive ability and moderating effects of emotional support

**Fig. 1:**
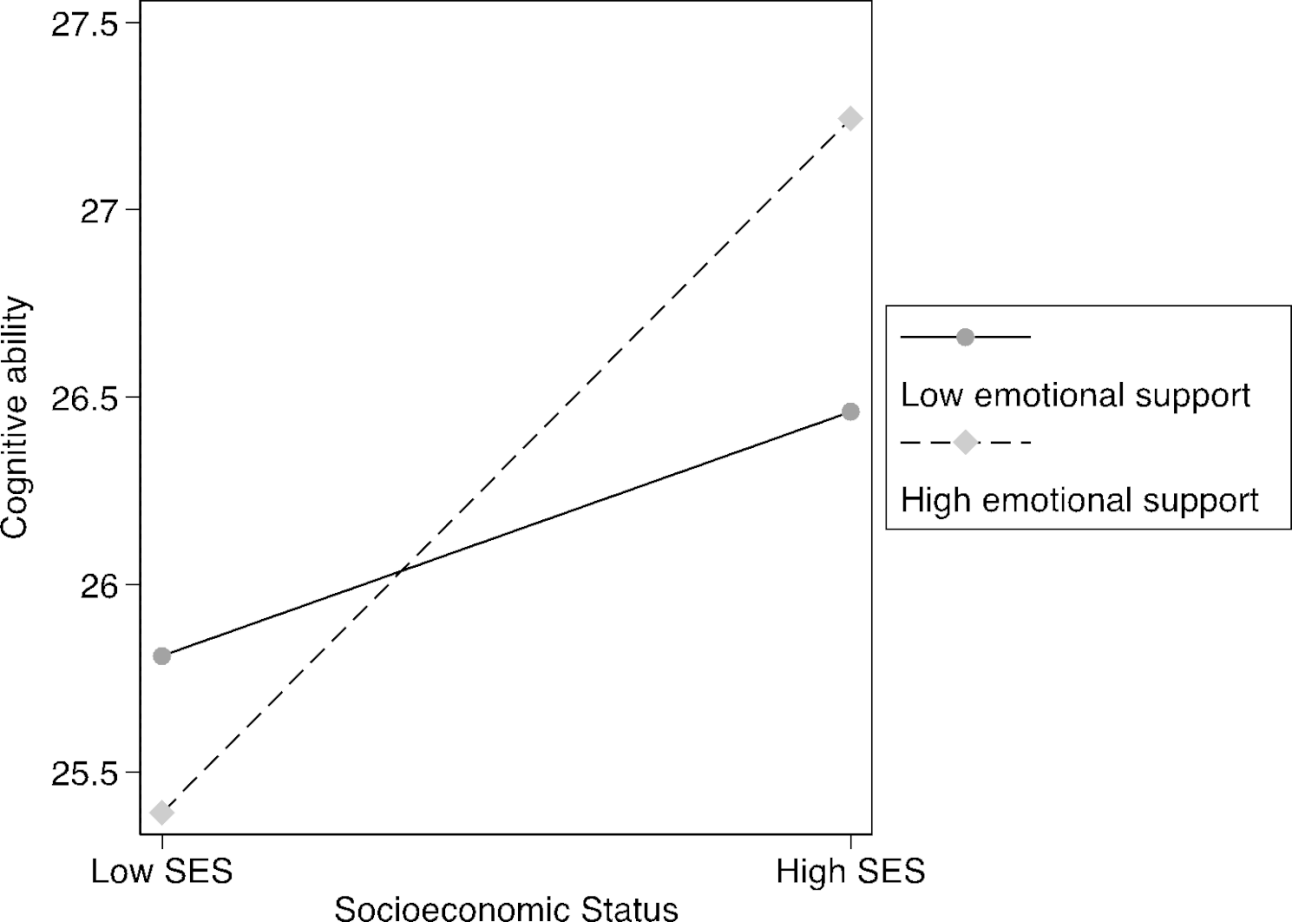
The moderating role of emotional support between SES and cognitive ability in the entire sample (N = 5,295)

Table 2 presents the results of a hierarchical analysis for the effects of SES on cognitive ability, and the moderating effects of emotional support in the relationship between SES score and cognitive ability among 5,295 participants. Model 1 included all sociodemographic variables as covariates. The results showed the participants who were married, having diet diversity, with normal BMI, and having health insurance were more likely to have better cognitive function. The increased age, female, rural residence, ADL and depression were negatively related to cognitive ability. Region, smoking, drinking, hypertension, diabetes, and the stroke were not associated with cognitive function. Model 2 further included SES in the model and showed higher SES score was positively associated with better cognitive function (β = 0.52, p < 0.001). Model 3 included emotional support and demonstrated it was not related to cognitive function when it treated as an individual factor. Model 4 showed emotional support was negatively related to cognitive function, and the interaction effect of SES and emotional support was significantly positive (β = 0.19, p < 0.001). That is, the result indicates a moderating effect of emotional support in the relationship between SES and cognitive ability, as visualized in Fig. 1. Although, the results showed more emotional support was associated with lower level of cognitive ability (β = -0.22, p < 0.01), the strength of the negative relationship between emotional support and cognitive ability was weaker for participants who have a higher SES status.

**Table 2:** A hierarchical analysis for moderating effects of emotional support in the relationship between socioeconomic status score and cognitive ability among 5,295 participants

	Model 1 β (95% CI)	Model 2 β (95% CI)	Model 3 β (95% CI)	Model 4 β (95% CI)
SES score		0.52^***^	0.51^***^	0.23^**^
		(0.38, 0.65)	(0.38, 0.65)	(0.05, 0.40)
Emotional support			0.07	-0.22^**^
			(-0.05, 0.20)	(-0.38, -0.05)
SES * Emotional support				0.19^***^
				(0.12, 0.26)
Age group	-1.64^***^	-1.59^***^	-1.60^***^	-1.58^***^
	(-1.77, -1.51)	(-1.72, -1.45)	(-1.73, -1.46)	(-1.71, -1.45)
Sex	-1.23^***^	-0.96^***^	-0.97^***^	-0.96^***^
	(-1.48, -0.97)	(-1.22, -0.70)	(-1.23, -0.70)	(-1.22, -0.70)
Marital status	0.28^*^	0.23	0.36^*^	0.37^*^
	(0.01, 0.56)	(-0.04, 0.50)	(0.01, 0.70)	(0.03, 0.72)
Region	0.07	0.08	0.08	0.07
	(-0.07, 0.22)	(-0.06, 0.22)	(-0.06, 0.23)	(-0.07, 0.21)
Hukou	-1.11^***^	-0.29	-0.3	-0.3
	(-1.39, -0.83)	(-0.64, 0.06)	(-0.65, 0.05)	(-0.65, 0.05)
Diet diversity	0.81^***^	0.71^***^	0.71^***^	0.72^***^
	(0.57, 1.05)	(0.47, 0.95)	(0.47, 0.95)	(0.48, 0.97)
Smoking	0.21	0.2	0.2	0.2
	(-0.11, 0.54)	(-0.13, 0.52)	(-0.13, 0.52)	(-0.13, 0.52)
Drinking	0.14	0.14	0.14	0.12
	(-0.18, 0.46)	(-0.18, 0.46)	(-0.18, 0.46)	(-0.20, 0.44)
BMI	0.37^**^	0.37^**^	0.37^**^	0.38^***^
	(0.14, 0.59)	(0.15, 0.60)	(0.15, 0.60)	(0.15, 0.60)
Exercise	0.30^*^	0.2	0.2	0.22
	(0.05, 0.54)	(-0.05, 0.44)	(-0.04, 0.45)	(-0.02, 0.47)
ADL	-1.17^***^	-1.18^***^	-1.18^***^	-1.18^***^
	(-1.30, -1.05)	(-1.30, -1.06)	(-1.30, -1.06)	(-1.30, -1.06)
Hypertension	0.24	0.21	0.21	0.21
	(-0.01, 0.48)	(-0.03, 0.44)	(-0.03, 0.44)	(-0.02, 0.44)
Diabetes	0.07	-0.01	-0.02	-0.02
	(-0.31, 0.46)	(-0.40, 0.37)	(-0.40, 0.37)	(-0.41, 0.36)
Stroke	-0.13	-0.15	-0.15	-0.2
	(-0.50, 0.25)	(-0.52, 0.23)	(-0.52, 0.23)	(-0.58, 0.17)
Health insurance	0.43^*^	0.45^*^	0.45^*^	0.44^*^
	(0.07, 0.79)	(0.09, 0.81)	(0.09, 0.80)	(0.09, 0.80)
Depression	-0.09^***^	-0.09^***^	-0.09^***^	-0.09^***^
	(-0.12, -0.07)	(-0.12, -0.06)	(-0.12, -0.06)	(-0.12, -0.06)
Constant	32.08^***^	29.68^***^	29.56^***^	30.04^***^
	(31.19, 32.98)	(28.59, 30.77)	(28.45, 30.67)	(28.92, 31.16)
*N*	5295	5295	5295	5295
Adjusted *R*^2^	0.3221	0.3291	0.3292	0.3326

Note: (1) 95% confidence intervals in brackets;

(2) * p < 0.05, ** p < 0.01, *** p < 0.001;

(3) All models were adjusted for a number of covariates, including age, sex, marital status, living region, hukou, health insurance, diet diversity, smoking, drinking, BMI, exercise, ADL, hypertension, diabetes, stroke or CVD, and depression

### The moderating effects of financial support in the relationship between SES score and cognitive ability

**Fig. 2:**
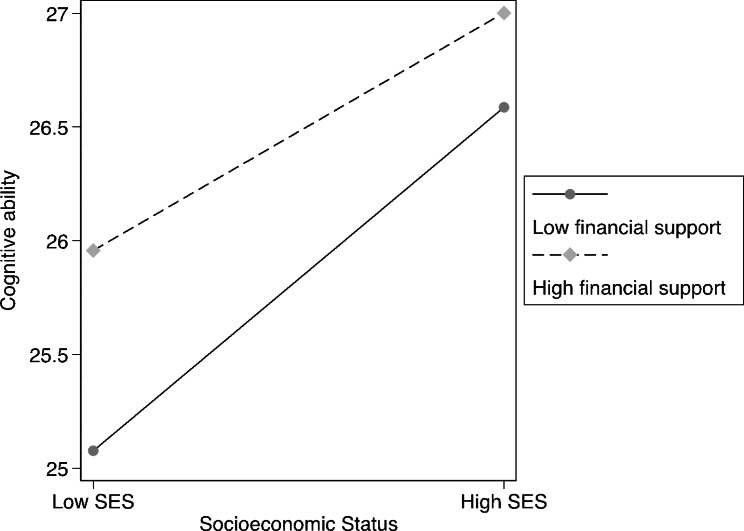
The moderating role of financial support between SES and cognitive ability in the entire sample (N = 5,295)

Table 3 presents the results of a hierarchical analysis for the effects of SES on cognitive ability, and the moderating effects of financial support in the relationship between SES score and cognitive ability. The results of Model 1 and Model 2 were the same as the results to the Table 2. Model 3 included financial support and it was positively associated with better cognitive ability (β = 0.08, p < 0.001). Model 4’s results indicate the interaction effect of financial support and SES is significantly negative (β = -0.03, p < 0.001), indicates a negative moderating effect of financial support in the relationship between SES and cognitive ability, as visualized in Fig. 2. The positive relationship between SES and cognitive ability was weaker for people who have a higher level of financial support.

**Table 3:** A hierarchical analysis for moderating effects of financial support in the relationship between socioeconomic status score and cognitive ability among 5,295 participants

	Model 1 β (95% CI)	Model 2 β (95% CI)	Model 3 β (95% CI)	Model 4 β (95% CI)
SES score		0.52^***^	0.54^***^	0.69^***^
		(0.38, 0.65)	(0.41, 0.68)	(0.50, 0.87)
Financial support			0.08^***^	0.13^***^
			(0.05, 0.12)	(0.08, 0.18)
SES * Financial support				-0.03^*^
				(-0.05, -0.00)
Age group	-1.64^***^	-1.59^***^	-1.57^***^	-1.57^***^
	(-1.77, -1.51)	(-1.72, -1.45)	(-1.70, -1.44)	(-1.70, -1.44)
Sex	-1.23^***^	-0.96^***^	-0.98^***^	-0.98^***^
	(-1.48, -0.97)	(-1.22, -0.70)	(-1.25, -0.72)	(-1.24, -0.71)
Marital status	0.28^*^	0.23	0.23	0.23
	(0.01, 0.56)	(-0.04, 0.50)	(-0.04, 0.50)	(-0.04, 0.50)
Region	0.07	0.08	0.09	0.09
	(-0.07, 0.22)	(-0.06, 0.22)	(-0.05, 0.24)	(-0.05, 0.24)
Hukou	-1.11^***^	-0.29	-0.35	-0.33
	(-1.39, -0.83)	(-0.64, 0.06)	(-0.70, 0.00)	(-0.69, 0.02)
Diet diversity	0.81^***^	0.71^***^	0.67^***^	0.66^***^
	(0.57, 1.05)	(0.47, 0.95)	(0.43, 0.91)	(0.42, 0.91)
Smoking	0.21	0.2	0.2	0.18
	(-0.11, 0.54)	(-0.13, 0.52)	(-0.13, 0.52)	(-0.14, 0.51)
Drinking	0.14	0.14	0.13	0.14
	(-0.18, 0.46)	(-0.18, 0.46)	(-0.19, 0.45)	(-0.18, 0.46)
BMI	0.37^**^	0.37^**^	0.37^**^	0.37^**^
	(0.14, 0.59)	(0.15, 0.60)	(0.15, 0.59)	(0.15, 0.60)
Exercise	0.30^*^	0.2	0.21	0.21
	(0.05, 0.54)	(-0.05, 0.44)	(-0.04, 0.45)	(-0.04, 0.45)
ADL	-1.17^***^	-1.18^***^	-1.18^***^	-1.18^***^
	(-1.30, -1.05)	(-1.30, -1.06)	(-1.30, -1.06)	(-1.30, -1.06)
Hypertension	0.24^*^	0.21	0.21	0.21
	(0.01, 0.48)	(-0.03, 0.44)	(-0.02, 0.44)	(-0.02, 0.44)
Diabetes	0.07	-0.01	-0.01	-0.01
	(-0.31, 0.46)	(-0.40, 0.37)	(-0.39, 0.37)	(-0.39, 0.37)
Stroke	-0.13	-0.15	-0.15	-0.14
	(-0.50, 0.25)	(-0.52, 0.23)	(-0.52, 0.23)	(-0.52, 0.23)
Health insurance	0.43^*^	0.45^*^	0.41^*^	0.41^*^
	(0.07, 0.79)	(0.09, 0.81)	(0.05, 0.76)	(0.06, 0.77)
Depression	-0.09^***^	-0.09^***^	-0.09^***^	-0.09^***^
	(-0.12, -0.07)	(-0.12, -0.06)	(-0.12, -0.06)	(-0.12, -0.06)
Constant	32.08^***^	29.68^***^	29.30^***^	29.00^***^
	(31.19, 32.98)	(28.59, 30.77)	(28.21, 30.40)	(27.87, 30.13)
*N*	5295	5295	5295	5295
Adjusted *R*^2^	0.3221	0.3291	0.3325	0.3329

Note: (1) 95% confidence intervals in brackets;

(2) * p < 0.05, ** p < 0.01, *** p < 0.001;

(3) All models were adjusted for a number of covariates, including age, sex, marital status, living region, hukou, health insurance, diet diversity, smoking, drinking, BMI, exercise, ADL, hypertension, diabetes, stroke or CVD, and depression

## Discussion

This study provided the national representative evidence about the direct impacts of SES on cognitive ability among Chinese older adults with considering several potential social support moderators. Overall, the results found that the SES was a significant predictor of Chinese older adults’ cognitive ability. Older people who got a disadvantaged socioeconomic status were more likely to develop a lower level of cognitive ability in later life. Moreover, we revealed that emotional support and financial support moderated the relationship between SES and cognitive ability.

Our study confirmed the direct effects of SES on Chinese older adults’ cognitive ability, which was consistent with the previous research conducted in other settings. For example, Zahodne et al. [29] found that adults with higher educational levels were associated with lower cognitive impairment at the later-life stage among American. This was because those well-educated people generally had high brain capacity than the people with lower educational attainment, giving them the advantage to slow down the deterioration in cognition. Similarly, scholars also found that individuals with better economic status and living conditions were associated with the higher cognitive ability [12, 18]. Compared with the existing evidence which largely focused on the single socioeconomic status, our results provided further evidence on the effect of a comprehensive SES score, including education, occupation, economic independence, and residence, on the older adults’ cognitive ability in China.

In addition to the direct association between SES and cognitive ability, this study demonstrated that social support served as an important moderator in such a relationship. Specifically, the main findings suggested that emotional support was a critical moderator that influenced the SES and cognitive ability among Chinese older adults, which was comparable with the previous study that emphasized the important role of emotional support [4]. Our finds was comparable with Zahodne and Watson et al., in which they found negative associations between emotional support and working memory were seen in Hispanics [42]. Interpretations of this negative association must take into account that this group exhibited lower cognitive performance had lower socioeconomic status. Thus, one potential explanation is reverse causation. Future longitudinal studies are needed to explore the possibility of reverse causation. Another potential explanation is that the emotional support-cognitive health relationship is non-linear, that means emotional support may be beneficial up to a point. Indeed, previous studies showed that excessive social support led to psychological distress and induced feelings of dependency [43]. Moreover, it is essential to consider the potation cultural and socioeconomic differences when studying the role of emotional support on cognitive ability among the older population.

Moreover, the positive relationship between SES and cognitive ability was weaker for people who have a higher level of financial support. Older adults who were in a disadvantaged SES typically received less financial support, which further made them more vulnerable to cognitive decline and mental problems [27]. This was corroborated with other studies which suggested that financial support had a greater impact on neuropsychiatric health among lower SES group subjects [8]. Indeed, there is some evidence that the effect of social support is varied health protective among difference SES groups [44]. Importantly, our results highlighted that the moderating roles of two types of social support were different in the relationship between SES and cognition. This was consistent with the previous findings that the association between social support and cognitive ability differed by sources [4]. Our finding also support the stress-buffering role of social support as a potential working mechanism, which are strongly associated with incident dementia [33, 35].

There are some limitations in this study. First, the cross-sectional design and data limited our understanding about the causal relationship between SES and cognitive ability. The reserves causality that difference in cognitive ability leads to the discrepancy of SES is also possible. Thus, further research should use more appropriate design and data to identify the causal link between SES and cognitive ability. Second, we only examined two type of potential moderator. However, the relationship between SES and cognitive ability is more complicated than we tested, indicating that there are many other possible pathways, such as cardio-metabolic and environmental risk factors. More research is needed to explore the underlying mechanisms that link SES and cognitive ability. Finally, the findings among the Chinese older adult sample may not necessarily be generalizable to other settings as we found the strong cultural effects in influencing the role of social support on cognitive ability. Further research could test the moderating role of social support in a different cultural context.

Despite these limitations, to our knowledge, this study contributes to the literature on SES inequalities in China. It confirms that SES serves as a significant factor that influences cognitive ability among Chinese older adults. In addition, to our knowledge, this is the first study that tests the moderating role of different types of social support on the link between SES and cognitive function. The findings of this study provide a nuanced understanding of SES inequalities on cognitive ability and highlight the importance of social support as a potential intervention to improve older adults’ mental health.

This study also contains important policy implications for improving older adults’ cognitive health conditions. Given that SES discrepancy is strongly associated with older adults’ cognitive ability, targeted efforts are needed to narrow disparities across different social groups, especially for those most vulnerable subpopulations, i.e., those with disadvantaged economic conditions, lower educational attainment, and poor living residence. In 2016, China’s government approved the Healthy China 2030 plan, which emphasized the need to strengthen effective interventions for the elder with cognitive impairment. More evidence-based and cost-effective approaches, such as digital technologies and tele-medicine, are needed to be utilized for early detection and intervention for the disease, that could have a dramatic effect on society. In addition, our results highlighted that the effects of social support differ by its source and type. In the Chinese context, policymakers should pay more attention to that family-oriented social support and use policy tools to encourage family members to have more contact with the elderly. Programs and policies that encouraging adults to live with or near their parents in order to take care of them in old age were issued in some provinces in China. Furthermore, participation in social activities is also recommended for older adults to increase social engagement and interaction, which further slows down the deterioration of cognitive ability.

## Conclusion

The current study found that SES was significantly associated with cognitive ability among Chinese older adults, and two types of social support moderated the relationship between SES and cognitive ability. These findings highlight the importance of narrowing the socioeconomic gap among the elderly in sustaining their cognitive ability in China. Policymakers should also consider promoting social support to improve the cognitive ability among older adults.

## Data Availability

The data that support the findings of this study are publicly available from the Peking University Open Research Data Platform.
